# Changes in trauma-related emergency medical services during the COVID-19 lockdown in the Western Cape, South Africa

**DOI:** 10.1186/s12873-023-00840-8

**Published:** 2023-06-27

**Authors:** Aleksandra Pettke, Willem Stassen, Lucie Laflamme, Lee Alan Wallis, Marie Hasselberg

**Affiliations:** 1 Department of Global Public Health, Karolinska Institutet, Stockholm, Sweden; 2 Division of Emergency Medicine, University of Cape Town, Cape Town, South Africa; 3 Institute for Social and Health Sciences, University of South Africa, Pretoria, South Africa

**Keywords:** Injuries, Physical trauma, COVID-19, Emergency Medical System, EMS, Lockdown, South Africa, Middle-income country

## Abstract

**Background:**

To limit virus spread during the COVID pandemic, extensive measures were implemented around the world. In South Africa, these restrictions included alcohol and movement restrictions, factors previously linked to injury burden in the country. Consequently, reports from many countries, including South Africa, have shown a reduction in trauma presentations related to these restrictions. However, only few studies and none from Africa focus on the impact of the pandemic restrictions on the Emergency Medical System (EMS).

**Methods:**

We present a retrospective, observational longitudinal study including data from all ambulance transports of physical trauma cases collected during the period 2019–01-01 and 2021–02-28 from the Western Cape Government EMS in the Western Cape Province, South Africa (87,167 cases). Within this timeframe, the 35-days strictest lockdown level period was compared to a 35-days period prior to the lockdown and to the same 35-days period in 2019. Injury characteristics (intent, mechanism, and severity) and time were studied in detail. Ambulance transport volumes as well as ambulance response and on-scene time before and during the pandemic were compared. Significance between indicated periods was determined using Chi-square test.

**Results:**

During the strictest lockdown period, presentations of trauma cases declined by > 50%. Ambulance transport volumes decreased for all injury mechanisms and proportions changed. The share of assaults and traffic injuries decreased by 6% and 8%, respectively, while accidental injuries increased by 5%. The proportion of self-inflicted injuries increased by 5%.

Studies of injury time showed an increased share of injuries during day shift and a reduction of total injury volume during the weekend during the lockdown. Median response- and on-scene time remained stable in the time-periods studied.

**Conclusion:**

This is one of the first reports on the influence of COVID-19 related restrictions on EMS, and the first in South Africa. We report a decline in trauma related ambulance transport volumes in the Western Cape Province as well as changes in injury patterns, largely corresponding to previous findings from hospital settings in South Africa. The unchanged response and on-scene times indicate a well-functioning EMS despite pandemic challenges. More studies are needed, especially disaggregating the different restrictions.

**Supplementary Information:**

The online version contains supplementary material available at 10.1186/s12873-023-00840-8.

## Background

In response to the COVID-19 pandemic, extensive physical distancing measures (“lockdowns”) were implemented in many countries. In South Africa, varying measures were implemented over time, including social distancing, curfews, restrictions on travel and movement and on alcohol sale.

The restrictions on travel and movement during the COVID-19 pandemic have led to a decrease in the amount of traffic collisions and consequently physical trauma presentations across the globe [1].

However, the reductions have not been uniform across the globe. There are disproportionalities in the reduction of traffic-related mortality and exposure leading to differences in affected road users, and changed patterns of injuries reported from both high-income (Australia [2], Greece [3], Finland [4], United Kingdom [5], United Arab Emirates [6]) and lower- middle-income countries (India [7], Nepal [8]).

In line with reduced movement due to pandemic restrictions, studies from several countries suggest a higher share of unintentional injuries in the domestic environment [9, 10]. Moreover, reports – especially the USA—suggest increased inter-personal violence resulting in an increase of shotgun or blunt and sharp trauma [11, 12].

Even though a high number of studies have been published on the topic, there are comparably few reports from low- and middle-income settings, even though in these countries the health burden from injuries is the highest.

Likewise, injuries and trauma have been a major public health concern in South Africa for a long time [13]. In 2019, injuries amounted to more than 11% of disability adjusted life years (DALYs) [14], meaning South Africa has one of the largest trauma burdens in the world. This is largely due to significant number of road traffic injuries and interpersonal violence [15]. In comparison in Gabon, another sub-Saharan upper-middle income country, injuries add up to less than 9% of DALYs [14], while in lower-middle-income country Ghana 7.24% of DALYs are estimated to be due to injuries [16].

The Western Cape is one of South Africa’s nine provinces and accounted for 11.8% of South Africa’s population in 2020 [17]. Previous data about injuries from the Western Cape suggests that more than 70% of patients injured are male and younger than 40 years of age. A majority of the trauma has been shown to be attributable to interpersonal violence (> 70%) and around traffic injuries (20%) [18]. Moreover, a clear pattern regarding injury time has been described with a greater share of intentional injuries occurring during the night and unintentional injuries during the day. Furthermore, two-thirds of all trauma has been described to occur on the weekend [19]. Alcohol has previously been identified as a major risk factor for intentional and unintentional injuries [20] and has been described to account for more than 30% of injuries in the Western Cape [18].

Following introduction of the virus by travellers returning from Europe, several different measures were combined in South Africa to tackle the COVID-19 pandemic, mainly modelling its response on measures being taken in Europe and on Europe’s estimates on its impact on society according to South African sources [21]. At the same time, these measures targeted factors tightly linked to injuries and trauma, like alcohol prohibition or restriction of movement. Therefore, this setting provided a unique opportunity to study their effect on trauma presentations. Studies from hospitals in several South African provinces have reported a similar trend as seen globally with admission reductions to emergency departments both in big urban settings [22, 23], and in rural areas [24], as well as a change in injury pattern during the lockdown [25]. However, previously published literature from South Africa comprises mostly small, single center reports from hospitals. Reports on a province level are lacking. Moreover, there are no reports from South Africa on Emergency Medical Service (EMS), and only very few reports on the influence of the pandemic on EMS worldwide. The aim of this study was to add to the knowledge of how restricted movement and alcohol sale affected the volume and nature of trauma presentations to the EMS in the Western Cape, South Africa.

## Methods

### Study design, setting and timeframe

The study presented is a retrospective, observational longitudinal study with data from all ambulance transports of physical trauma cases collected from the Western Cape Government Emergency Medical Service (WCG EMS) in all of the Western Cape Province, South Africa between 2019–01-01 and 2021–02-28. During the study period, different restrictions were put in place in different times of the pandemic. The restrictions followed a five level system with strictest restrictions in Lockdown Level 5 and least strict in Lockdown Level 1 (for a detailed description of measures in each level see Supplementary Table 1).

Ambulance transport volumes before and during the pandemic were compared. Data were analysed divided into three timeframes, each 35-day long:

The lockdown-period: Lockdown Level 5 imposed by the South African government (27 March to 30 April 2020, 35 days).

Pre-lockdown period: 35 days prior to lockdown (21 February to 26 March 2020, 35 days).

Pre-COVID-19-period: corresponding timeframe to lockdown Level 5 in 2019, the year before the COVID-19 pandemic (27 March to 30 April 2019, 35 days).

The Western Cape province has a formalised EMS system [26] that is staffed by emergency care providers that have basic to advanced scopes of practice. Each ambulance is staffed by two emergency care providers with the option of clinical support from an advanced provider. Training for emergency care providers range from six weeks to four years. WCG EMS transports patients to 34 districts, four regional, and three central public hospitals in the region.

### Data collection and management

After every patient encounter, an emergency care provider is required to complete an electronic patient care record (ePCR). Similarly, every request for an emergency response is recorded on the WCG EMS computer-aided dispatch (CAD) system. These two systems were the sources of data to minimise missingness. Access to the WCG EMS database provided a comprehensive dataset for this study, allowing to study the effect of restrictions on EMS province-wide for the first time.

Electronic data were extracted from the databases and anonymised prior to data collection. All patients with injury complaints that were responded to during the study period were included. All injuries were included regardless of intent or mechanism of injury. Duplicates and cancelled emergency calls were excluded prior to analysis.

Furthermore, due to a moratorium on research by private emergency services during the COVID-pandemic and largely paper-based records, data from private ambulance providers was not complete and emergency calls responded to by private ambulance providers were excluded prior to analysis. Research was confined to care provided by the provincial EMS, which amounts to more than 75% of the trauma response in the Western Cape province.

### Variables

Variables related to injury characteristics were studied in detail (injury intent, injury mechanism and injury severity based SATS triage by ambulance staff on scene). To facilitate analysis of injury mechanism, the initially provided granular classifications of injury mechanisms were grouped into broader injury mechanisms and by intent (Supplementary Table 2).

Furthermore, time of injury was investigated, both regarding time of day and day of the week. On-scene-times and call-to-arrival-times were calculated by aggregating the time when the emergency call was made and the time when the ambulance arrived on scene and left the scene, respectively. Sociodemographic variables provided were patient age and gender.

### Statistical analysis

Descriptive analysis of the data was performed. The volume of ambulance transports over time was determined by total numbers and as share of the total (%). The dataset has been tested for normality using a Shapiro–Wilk test. The analysis confirmed dataset normality of the dataset on injury intent, mechanism and severity as well as time of injury (day/night – weekday/weekend). Chi square analysis was performed to determine the significance of the differences detected between different time periods as described previously. The following comparisons were made:

1. Lockdown period – Pre-lockdown period
2. Lockdown period – Pre-COVID-19-period
3. Pre-lockdown period – Pre-COVID-19-period

Normality analysis using a Shapiro–Wilk test revealed non-parametricity of the data on “time to scene arrival” and “ambulance on-scene-time”. Consequently, a Kruskal–Wallis test was used to determine significance of the differences detected between different time periods as described previously.

## Results

### Description of the dataset

Altogether, the sample size for the whole sampling period was 103,250 cases. Adults over 18 and below 105 years of age with a defined mechanism of injury and valid time of ambulance arrival were included in the study, resulting in 87,167 cases.

From 1st January 2019 until 26th March 2020 when COVID-related restrictions were imposed, there were 58,508 cases, and from introduction of COVID-19 related restriction until 28th February 2021 there were 28,659 trauma cases.

Overall distribution of calls between day and nightshift was even. More than 50% of injuries were transported during the weekend (27.1% on a Saturday and 25.6% on a Sunday). During the whole study period, most cases were triaged as severe injuries (orange and red (52.6%)). Most transported cases were intentional injuries (64.2%). The most common reported injury mechanism was assault (56.4%), followed by traffic injuries (16.7%) and accidental injuries (14%). A majority of reported patients was men (65.4%) and between 31 and 64 years old (51.9%) (Supplementary Table 3).

### Influence of the restrictions due to the COVID-19 pandemic on overall trauma volume, injury mechanism, and severity of injuries

An analysis of the number of transported trauma cases in relation to time of restriction revealed a reduction by more than 50% in April 2020, the first month after introduction of restrictions due to the COVID-19 pandemic in South Africa (Supplementary Fig. 1). Over the course of 2020 until the end of the analysis period the trend in trauma cases largely followed the level of restrictions. As restrictions were gradually reduced, the number of cases increased and reached a plateau between October and December 2020. In January 2021, the number of trauma cases dropped again by one third as restrictions were tightened followed by a 50% increase during January and February 2021 (Supplementary Fig. 1).

Next, injury mechanisms were stratified according to intent and injury mechanism to examine the changes in trauma volume in more detail. Over the whole study period, intentional injuries accounted for a majority of trauma cases in the Western Cape (Supplementary Fig. 2). The shares of intentional and unintentional injuries of the total number of injuries stayed stable over time. While the share for reports of intentional injuries undulated around 60% and the share of unintentional injuries undulated around 40%, the proportion between intent was not influenced by restrictions (Supplementary Fig. 2).

**Fig. 1:**
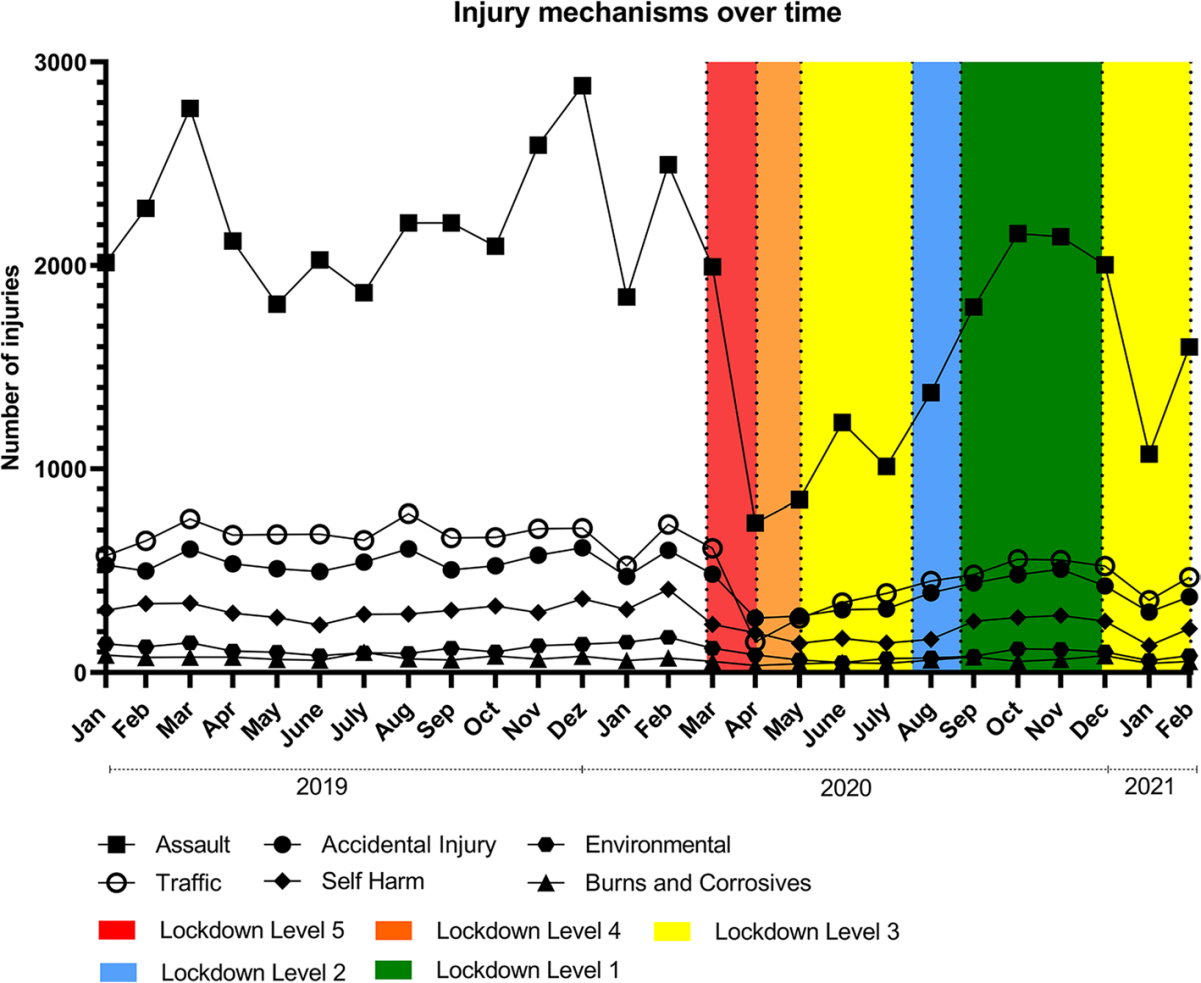
Total numbers of different injury mechanisms over the study period

The total number of reported intentional and unintentional injuries largely followed the pattern detected for total number of trauma cases. The introduction of lockdown Level 5 measures on 27 March 2020 was accompanied by a reduction of intentional and unintentional injuries by *ca.* 40% each which was not statistically significant (Table 1). Investigations of the course of the reported injury mechanism over time revealed a decrease of case-numbers in all injury mechanisms, while the proportions changed significantly during Lockdown Level 5 compared to pre-lockdown and the same time period in 2019 (*p* < 0.05, Fig. 1, Table 1). The share of assaults decreased by 5.6% during lockdown level 5 compared to both pre-lockdown periods and the share of traffic injuries by 7% compared to 2019 and 7.9% compared to the immediate pre-lockdown period. Accidental injuries increased by 4.7% during the lockdown level 5 compared with pre-lockdown. The share of environmental injuries and injuries by burns and corrosives doubled during the pandemic lockdown. The proportion of self-inflicted injuries also increased markedly from 7.1% in the pre-lockdown period to 12.5% during lockdown level 5.

**Table 1:** Descriptive analysis of injury intent, mechanism and severity in three different periods. Significance between indicated periods was determined using Chi square analysis

Variables	2019		Pre-lockdown		Lockdown Level 5		*p*-value		
							Lockdown – Pre-lockdown	Lockdown—2019	Pre-lockdown—2019
	*n* = 4706 (%)		*n* = 4587 (%)		*n* = 1806 (%)				
**Injury Intent**									
Intentional Injuries	3004	(64.1%)	2914	(63.6%)	1140	(63.4%)	0,85	0,58	0,62
Unintentional Injuries	1682	(36.9%)	1667	(36.4%)	659	(36.6%)			
**Injury mechanism**									
Accidental	650	(13.9%)	617	(13.5%)	327	(18.2%)	< 0.05	< 0.05	0.16
Assault	2648	(56.5%)	2589	(56.5%)	915	(50.9%)			
Burns and Corrosives	89	(1.9%)	63	(1.4%)	43	(2.4%)			
Environmental	132	(2.8%)	152	(3.3%)	104	(5.8%)			
Self-Harm	356	(7.6%)	325	(7.1%)	225	(12.5%)			
Traffic	811	(17.3%)	835	(18.2%)	185	(10.3%)			
**Triage**									
Blue	53	(1.2%)	62	(1.4%)	23	(1.3%)	0.54	< 0.05	< 0.05
Mild (green & yellow)	3445	(74.9%)	3450	(76.9%)	1338	(75.7%)			
Severe (orange & red)	1102	(24.0%)	974	(21.7%)	406	(23.0%)

**Fig. 2:**
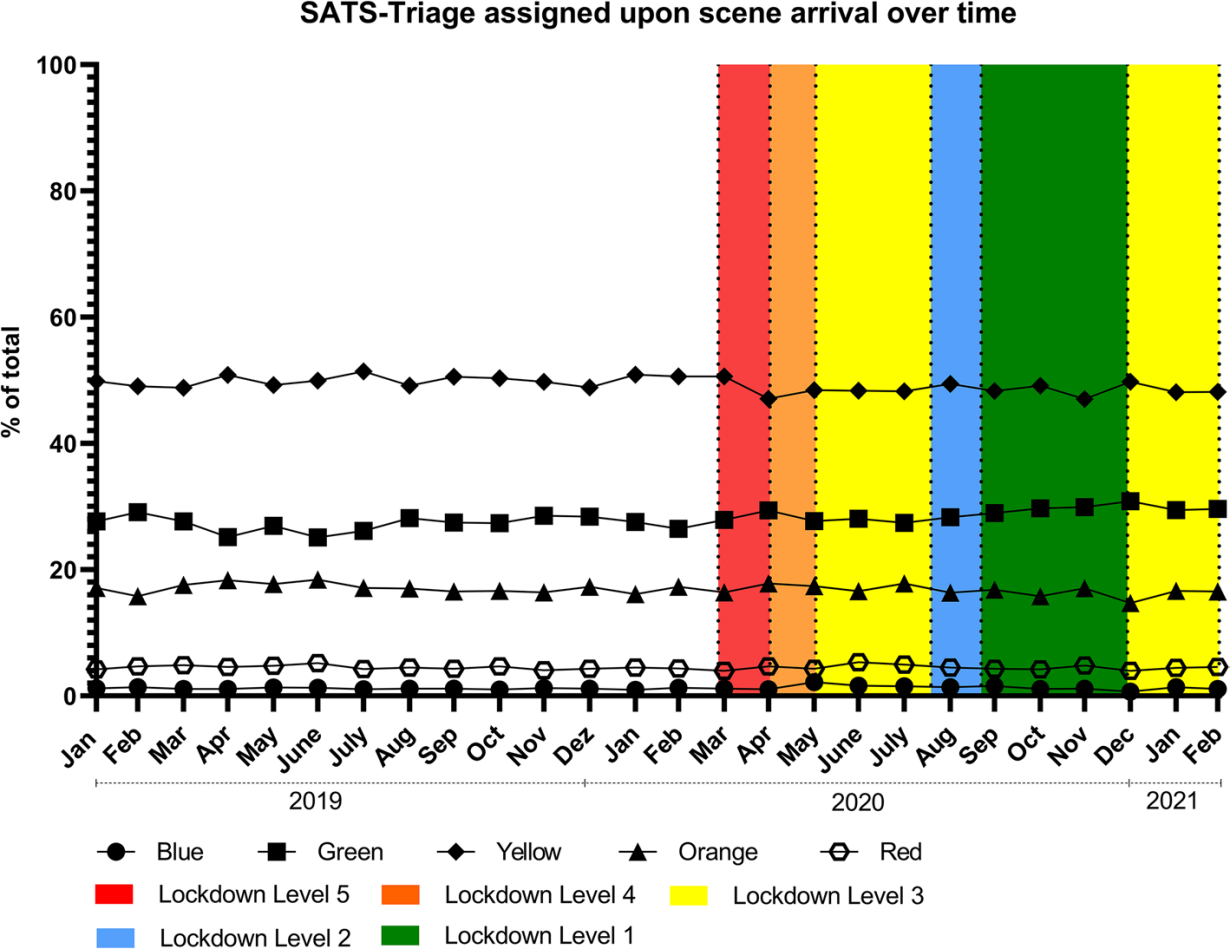
Share of different injury-severity levels over the study period determined by SATS-triage by ambulance staff upon scene arrival

To further assess changes in the nature of injuries during the pandemic, on-site triage by ambulance staff was assessed as a marker for case severity. The triage of the injury cases stayed stable across the whole study time (Fig. 2), undulating 1% around 76% for mildly injured (green and yellow), and 1% around 23% in triaged as severely injured (orange and red) in all study periods (Table 1).

### Changes in injury time both regarding time of day and weekdays

Next, changes to the time of injury report in the three different periods 2019, Pre-lockdown and Lockdown Level 5 were analysed. The time between 6am and 6 pm was counted as day, and between 6 pm to 6am as night.

With the implementation of Lockdown Level 5 measures in week 13 in 2020, the share of injuries reported during day increased from 47.0% to 57.9%, and the share of injuries during night-time decreased from 53.0% to 42.1% (*p* < 0.05, Table 2). As seen with the total number of injuries, the change in the pattern when injuries happened, largely following the level of restrictions (Supplementary Fig. 3). During less strict measures, the share of injuries happening in night-time increased and upon re-introduction of more strict measures, it decreased again.

**Table 2:** Descriptive analysis of injury time regarding time of day and part of the week in three different periods. Significance between indicated periods was determined using Chi square analysis

Variables	2019		Pre-lockdown		Lockdown Level 5		*p*-value		
							Lockdown – Pre-lockdown	Lockdown—2019	Pre-lockdown—2019
**Total number of injuries**	*n* = 4706 (%)		*n* = 4587 (%)		*n* = 1806 (%)				
**Time of day**									
Day	2282	(48.5%)	2155	(47.0%)	1046	(57.9%)	< 0.001	< 0.001	0.145
Night	2424	(51.5%)	2432	(53.0%)	760	(42.1%)			
**Part of the week**									
During the week	2138	(45.4%)	1943	(42.4%)	1171	(64.8%)	< 0.001	< 0.001	< 0.05
Weekend	2568	(54.6%)	2644	(57.6%)	635	(35.2%)			

An analysis of the day of the week during which the cases were reported, revealed a shift of the number of injuries from a clear focus on weekend-days, to a more even distribution on all weekdays (Table 2). In relation to the whole week, reports of injuries during weekend days declined by more than 15% for both Saturday and Sunday, respectively, during lockdown Level 5 (*p* < 0.05, Table 2) as compared to the immediate pre-lockdown period and the same period in 2019, respectively.

### Changes in time to scene arrival and ambulance on-scene-time

To study the performance of the WCG EMS during the pandemic, the time it took ambulances to arrive and the on-scene time were studied. The median time remained stable (*p* = 0.8727) for both response-time as well as on-scene-time in the time-periods studied (Table 3).

**Table 3:** Median times to ambulance arrival and on-scene-times in minutes

	**Minutes to ambulance arrival**			**Ambulance on-scene-time**		
	Median	IQR		Median	IQR	
**2019**	15	9	28	12	7	20
**Pre-Lockdown**	16	9	28	12	7	20
**Lockdown**	15	9	26	12	6	20

## Discussion

To our knowledge, this is the first study from South Africa based on such a large dataset from a province-wide geographical catchment area, allowing for a comprehensive study of trauma in a whole South African province in the context of the pandemic. Importantly, only few studies looked at EMS during the COVID-19 pandemic, and none of these studies was based in (South) Africa. Additionally, the extensive WCG EMS database provides information on many different aspects of the reported injuries, permitting to study many different aspects of physical injuries including the time, intent, and ambulance service times.

Our study revealed a decreased total number of injuries, especially at the beginning of the restrictions. These results confirm previous studies from South Africa and other parts of the world [1], reporting a decrease in trauma presentations during the COVID-19 pandemic. The extent of reduction of about 50% during Lockdown Level 5 seen in this report is in line with previous reports from different South African provinces like the Western Cape [23], KwaZulu Natal [24] and Gauteng [22, 28].

Furthermore, one of the key findings is a change of proportions in the trauma cases: While there was a marked decrease in the proportion of traffic injuries and assaults, the share of self-harm and environmental injuries increased. Studies from hospitals in different regions in South Africa, including the Western Cape, point in a similar direction [29]. Mental health conditions have been reported as one of the sequelae of the pandemic restrictions in many countries around the world. First reports from Africa suggest that almost 40% of African adults suffered from anxiety symptoms during the COVID-19 pandemic [30]. Furthermore, the pandemic has been shown to disproportionally increase violence against women [31], and thus increase mental health problems in women [32]. These first studies may provide an explanation for not observing a decrease in injuries resulting from self-harm in this study. However, the true dimensions of the pandemic’s impact on mental health remain to be studied.

Notably, there are also contrasting reports from the Western Cape [33] of unchanged proportions of different types of trauma. These differing findings can be due to regional differences in socio-demographic factors of the catchment areas of the study-hospitals and highlight the value of this study providing a province-wide overview. Moreover, the divergent findings can be rooted in different data sources, since Mahoney et al. [33] are based on hospital data and this study focuses on EMS data.

To our knowledge, this is the first report looking at injury severity in South Africa during the COVID-pandemic. Interestingly, the pattern of SATS triage did not follow the same drastic changes as injury volume. The changes in the pattern of triaging between pre-lockdown and lockdown period were slight with only 1% increase in severe cases and 1% decrease in mild cases, respectively. This finding contrasts with reports on decreased case numbers, but increased injury severity from European countries [34, 35] as well as Rwanda [36]. Previous reports from South Africa have shown severe under-triage in pre-hospital emergency services, where 29.5% received a too low triage score mainly due to incorrectly selected clinical discriminators[37]. Taking into account this information and the discrepancy to reports from other countries with increased severity of injuries during the pandemic, the lack of increase in severity might indicate a resource-constrained pre-hospital care system function during the pandemic, where the staff potentially lack time to correctly assess the clinical condition of the trauma patients.

However, the surprisingly stable response- and on-scene-times in the time-periods studied contradict a potential resource-constraint. In case of resource constraint or high pressure, prolonged response- and on-scene-times could be expected. Moreover, additional measures taken during the pandemic like putting on personal protective equipment might also be expected to increase response- and on-scene-times. Hardcastle et al. report pro-longed pre-hospital time due to EMS-shortages, including sick personnel as well as ambulances being used for COVID-patients [38]. The lack of an increase reported in this study suggests a good functionality of the pre-hospital emergency care systems even during the increased pressure in the pandemic. However, this study did not systematically collect data on health care quality. More research linking pre-hospital emergency services and hospital care will be needed to elucidate the actual case severity during the pandemic. Such a universal analysis of the EMS and hospital services, might provide valuable insights into the functioning of the WCG EMS and potential ways to improve the use of resources in emergency care.

Completing the data collected with actual injury severity data collected from hospitals might provide valuable insights into the functioning of the WCG EMS and potential ways to improve the use of resources in pre-hospital emergency care.

Furthermore, we have reported an increased percentage of injury reports during day shift compared to night shift during lockdown Level 5, as well as a shift from an injury-focus on the weekend to weekdays. We assume that the change we see could be a result of the reduced exposure due to a curfew between 8 pm and 5 am imposed on the population of the Western Cape during the lockdown Level 5. Unfortunately, the dataset used in the study does not contain information about potential exposure to injury. Data modelling of mobility in South Africa during the COVID-10 pandemic, suggests reductions in mobility of up to 78% during Lockdown Level 5 [39], however the data does not discriminate time of mobility. To our knowledge no other studies have investigated time of injury during the COVID-19 related lockdowns.

A key strength of this study is its large dataset comprised of more than 87,000 cases from the whole of the Western Cape. Previously published studies about the influence of the COVID-19 pandemic on trauma burden in South Africa were based on small datasets of only a few hundred to thousand cases [23, 27, 31] with limited geographical catchment areas. Moreover, previously published reports from South Africa were facility based studies, and did not study EMS use and performance during the pandemic.

Another advantage of the dataset used, is its large amount of information on many different aspects of the injury reported including injury intent, mechanism, severity measured by SATS triage, and time distribution, allowing a broad overview of the impact of COVID-19 induced restrictions on injuries in the Western Cape.

However, there are also inherent limitations to the study performed. An important limitation of this study is missing disaggregation of measures taken during the Lockdown Level 5 period. However, several different measures were taken simultaneously restricting both movement and alcohol sale. Each of the measures is likely to have an effect on the burden of injuries, and their interplay and influence on each other is complex. It is unclear how independent the different restrictions are in their impact on trauma burden making a true disaggregation difficult. Furthermore, additional factors, like fear of COVID-19 infection or socio-economic status, might be influencing aspects that were not accounted for.

## Conclusions

We report a reduction in the total number of trauma cases transported by the WCG EMS by 62% during the lockdown level 5 period compared to the same timeframe in 2019 as well as a change in the proportion of different trauma mechanisms.

The unchanged injury severity as determined on-site by SATS triage as well as the unchanged ambulance response times are particularly interesting study-results since they can be seen as functionality-indicators of the WCG EMS during a pressured time.

All in all, the data presented here will deepen the understanding of the influence of restrictions to alcohol sale and movement on both trauma burden and pre-hospital emergency care. It is the first study from South Africa to present province-wide data with information on many aspects of injuries, and in particular, it is the first study to present EMS data from South Africa during pandemic times.

## Supplementary Information

**Additional file 1.** Supplementary Material containing: Figures S1-S3.

**Additional file 2.** Supplementary Tables 1—3.

## Data Availability

Data were available electronically from the Computer Assisted Dispatch (CAD) system at the WCG EMS. They represent government EMS data and therefore are not shared here. The datasets generated and/or analysed during the current study are not publicly available due to the reasons stated above, but are available from the corresponding author on reasonable request.

## Abbreviations

WCG EMS: Western Cape Government Emergency Medical Service
CAD: Computer Assisted Dispatch
ePCR: Electronic patient care record
