# Supplementary figures and images for: Changes in trauma-related emergency medical services during the COVID-19 lockdown in the Western Cape, South Africa
Source: BMC Emerg Med. 2023 Jun 27;23:72. doi: 10.1186/s12873-023-00840-8 (PMC10304331; doi:10.1186/s12873-023-00840-8)

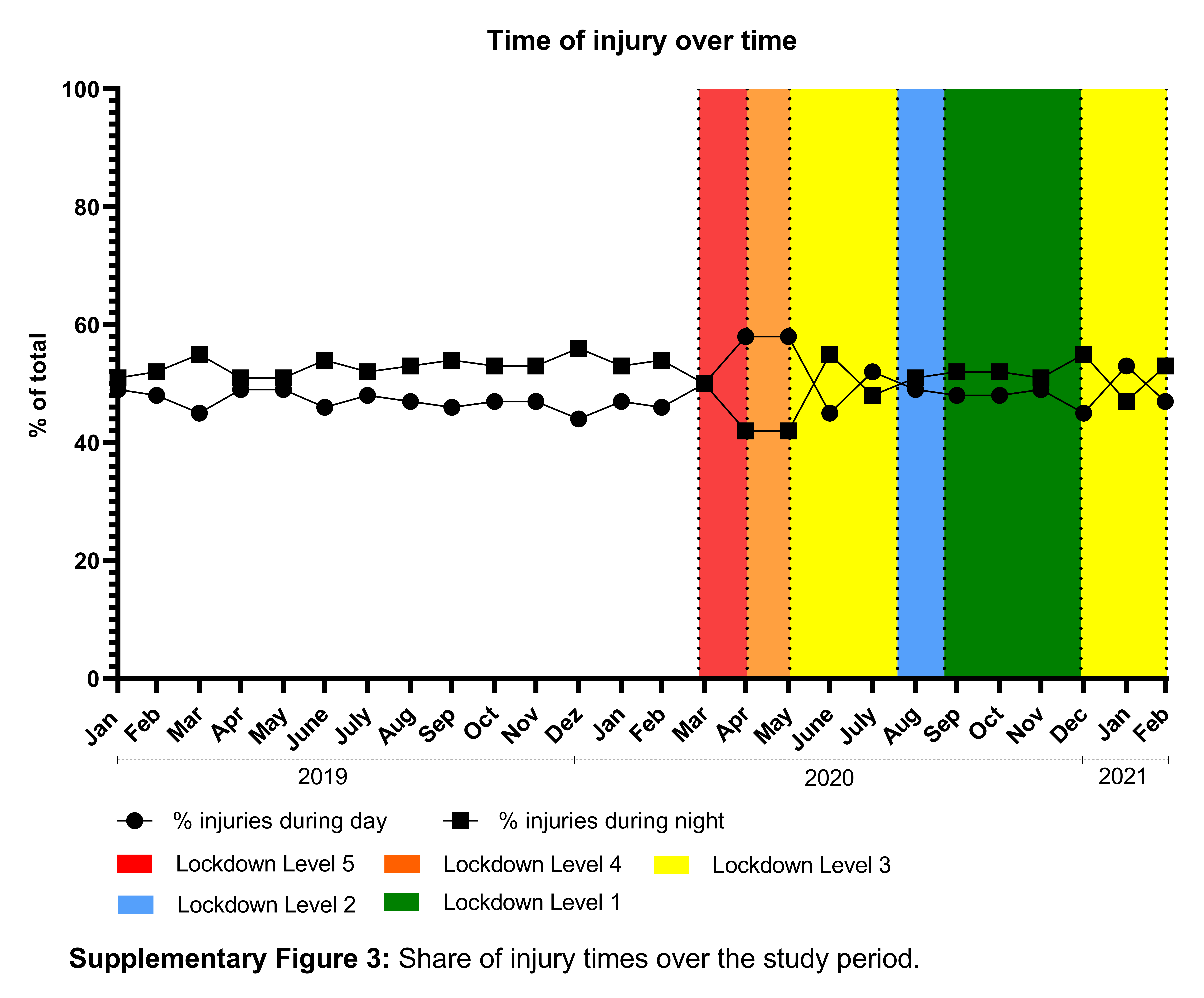

Supplement: Supplementary file 1 — Additional file 1. Supplementary Material containing: Figures S1-S3. [file 12873_2023_840_MOESM1_ESM.zip › Pettke et al_Supplementary Figure 3.tif]
