# Supplementary material for: Changes in trauma-related emergency medical services during the COVID-19 lockdown in the Western Cape, South Africa
Source: BMC Emerg Med. 2023 Jun 27;23:72. doi: 10.1186/s12873-023-00840-8 (PMC10304331; doi:10.1186/s12873-023-00840-8)
Supplement: Supplementary file 2 — Additional file 2. Supplementary Tables 1—3. [file 12873_2023_840_MOESM2_ESM.docx]

Pattern of trauma presentations during the COVID-19 lockdown in the Western Cape, South Africa

Supplementary Material

**Supplementary table 1**. Overview of Lockdown levels and associated restrictions during relevant timeframe

| **Lockdown** | **Dates Effective** | **Summary of Restrictions** |
| --- | --- | --- |
| **None** | Before 2020-03-27 | - No restrictions |
| **Level 5** | 2020-03-27 – 2020-04-30  (35 days) | - Only essential travel - No interprovincial travel - All non-essential commercial and industrial activities suspended - Curfew 20h00 – 05h00 - No public gatherings - No alcohol sales |
| **Level 4** | 2020-05-01 – 2020-05-31  (31 days) | - Only essential travel - No interprovincial travel except for special circumstances (e.g. funerals) - All non-essential commercial and industrial activities suspended - Curfew 20h00 – 05h00 - Public gatherings limited to 50 people - Restaurants open for take-away meals - No alcohol sales |
| **Level 3** | 2020-06-01 – 2020-08-17  (77 days) | - Interprovincial travel for business permitted - All commercial and industrial activities permitted - Curfew 21h00 – 04h00 - Public gatherings for worship, auctions or funerals permitted - Alcohol sales Mon to Thurs 09h00 - 17h00 |
|  | 2020-07-13 – 2020-08-17 | - Restrictions extended by: No alcohol sales |
| **Level 2** | 2020-08-18 – 2020-09-20  (33 days) | - Most restrictions lifted - Interprovincial travel permitted - Most restrictions lifted - Curfew 22h00 – 04h00 - Any public gathering up to 50 people - Alcohol sales for off-site consumption Mon to Thurs 09h00 - 17h00 |
| **Level 1** | 2020-09-21 – 2020-12-28  (98 days) | - All domestic travel permitted - All economic activities permitted - Curfew 00h00 – 04h00 - Alcohol sales for off-site consumption permitted Mondays to Fridays from 09h00 until 17h00; on-site consumption Mondays to Sundays until curfew. |
| **Level 3** | 2020-12-29 – 2021-02-28  (62 days) | - Interprovincial travel for business permitted - All commercial and industrial activities permitted - Public gatherings for worship, auctions or funerals permitted - Curfew 23h00 – 04h00 - Alcohol sales Mon to Thurs 10h00 - 18h00 |

**Supplementary Table 2:** Grouping of injury mechanisms by injury intent and form

| Original Injury Forms | Grouped by injury form | |
| --- | --- | --- |
| **Unintentional** |  | |
| Accidental Injury - Domestic | Accidental injury | |
| Accidental Injury - Industrial | Accidental injury | |
| Accidental Injury - Sports | Accidental injury | |
| Aircraft | Traffic | |
| Burns and Corrosives | Burns and Corrosives | |
| Bus / Taxi | Traffic | |
| Cyclist | Traffic | |
| Electrocution | Environmental | |
| Environmental - Bites and Stings | Environmental | |
| Environmental - Cold Exposure | Environmental | |
| Environmental - Heat Exposure | Environmental | |
| Hazmat Transport Incident | Traffic | |
| Light Motor Vehicle | Traffic | |
| Motorcyclist | Traffic | |
| Near Drowning | Environmental | |
| Pedestrian | Traffic | |
| Train and Railway Incident | Traffic | |
| Transport (Other) | Traffic | |
| Truck / Heavy Vehicles | Traffic | |
| Watercraft | Traffic | |
| **Intentional** |  | |
| Assault - Physical | Assault | |
| Assault - Poisoning | Assault | |
| Assault - Sexual | Assault | |
| Assault - Weapon (Gunshot) | Assault | |
| Assault - Weapon (Other) | Assault | |
| Self-Harm - Other | Self-Harm | |
| Self-Harm - Poisoning | Self-Harm | |
| Self-Harm - Weapon (Gunshot) | Self-Harm | |
| Self-Harm - Weapon (Other) | Self-Harm |  |
| **Unclear mechanism - excluded from analysis** | | |
| Bleeding | Unclear mechanism | |
| CPR in Progress | Unclear mechanism | |

**Supplementary Table 3:** Summary of cases during the whole study period grouped by variables

|  | **Number of cases** | **% of total** |
| --- | --- | --- |
| **Day of the week** |  |  |
| Monday | 9012 | 10,3 |
| Tuesday | 7691 | 8,8 |
| Wednesday | 7307 | 8,4 |
| Thursday | 7267 | 8,3 |
| Friday | 10005 | 11,5 |
| Saturday | 23596 | 27,1 |
| Sunday | 22289 | 25,6 |
| **Total all weekdays** | **87167** | **100,0** |
|  |  |  |
| **Triage** |  |  |
| Blue | 1140 | 1,3 |
| Green | 23832 | 27,3 |
| Yellow | 14608 | 16,8 |
| Orange | 4089 | 4,7 |
| Red | 41745 | 47,9 |
| missing values | *1753* | *2,0* |
| **Total** | **87167** | **100,0** |
|  |  |  |
| **Injury intent** |  |  |
| intentional | 55978 | 64,2 |
| unintentional | 31144 | 35,7 |
| missing values | *45* | *0,1* |
| **Total** | **87167** | **100,0** |
|  |  |  |
| **Injury Mechanism** |  |  |
| Accidental Injury | 12178 | 14,0 |
| Assault | 49166 | 56,4 |
| Burns and Corrosives | 1682 | 1,9 |
| Environmental | 2707 | 3,1 |
| Self Harm | 6812 | 7,8 |
| Traffic | 14577 | 16,7 |
| missing values | *45* | *0,1* |
| **Total** | **87167** | **100,0** |
|  |  |  |
| **Gender** |  |  |
| Female | 28658 | 32,9 |
| Indetermined | 34 | 0,0 |
| Male | 57035 | 65,4 |
| Unknown | 923 | 1,1 |
| missing values | *517* | *0,6* |
| **Total** | **87167** | **100,0** |
|  |  |  |
| **Age** |  |  |
| Young adult (18-30) | 38455 | 44,1 |
| Adult (31-64) | 45222 | 51,9 |
| Oder person (65+) | 3441 | 3,9 |
| missing values | *49* | *0,1* |
|  | **87167** | 100,0 |
